# Successful and Less Successful Psychotherapies Compared: Three Therapists and Their Six Contrasting Cases

**DOI:** 10.3389/fpsyg.2019.00816

**Published:** 2019-04-17

**Authors:** Andrzej Werbart, Amanda Annevall, Johan Hillblom

**Affiliations:** Department of Psychology, Stockholm University, Stockholm, Sweden

**Keywords:** unsuccessful treatments, non-improvement, negative processes, therapeutic relationship, patient and therapist perspective, outcome and process research, qualitative research methods, psychoanalytic psychotherapy

## Abstract

Despite the general effectiveness of bona fide psychotherapies, the number of patients who deteriorate or fail to improve is still problematic. Furthermore, there is an increased awareness in the field that the therapists’ individual skills make a significant contribution to the variance in outcome. While some therapists are generally more successful than others, most therapists have experienced both therapeutic success and failure in different cases. The aim of this case-series study was to deepen our understanding of what matters for the therapists’ success in some cases, whereas other patients do not improve. How do the patients and their therapists make sense of and reflect on their therapy experiences in most successful and unsuccessful cases? Are there any distinctive features experienced by the participants at the outset of treatment? To explore these issues, we applied a mixed-method design. Trying to keep the therapist factor constant, we selected contrasting cases from the caseloads of three therapists, following the criterion of reliable and clinically significant symptom reduction or non-improvement at termination. Transcripts of 12 patient interviews and 12 therapist interviews (at baseline and at termination) were analyzed, applying inductive thematic analysis and the multiple-case comparison method. The comparisons within the three therapists’ caseloads revealed that in the successful cases the patient and the therapist shared a common understanding of the presenting problems and the goals of therapy and experienced the therapeutic relationship as both supportive and challenging. Furthermore, the therapists adjusted their way of working to their patients’ needs. In non-improved cases, the participants presented diverging views of the therapeutic process and outcome. The therapists described difficulties in the therapeutic collaboration but not how they dealt with obstacles. They tended to disregard their own role in the interactions and to explain difficulties as being caused by the nature of their patients’ problems. This could indicate that the therapists had difficulty in reflecting on their own contributions, accepting feedback from their patients, and adjusting their work accordingly. These within-therapist differences indicate that taking a “third position” is most needed and seems to be most difficult, when early signs of a lack of therapeutic progress appear.

## Introduction

Most psychotherapy research focuses on the validation of treatment effects for patients with various psychological problems. However, psychotherapy is not always helpful. While the general effectiveness of bona fide psychotherapies is well established, the number of patients who fail to improve or even deteriorate is still problematic (Hansen et al., 2002; Lambert, 2007, 2011, 2013; Warren et al., 2010). Failure in psychotherapy is a complex topic, and the term has been used for a broad array of disparate unwanted effects, such as attrition, non-response, deterioration, adverse outcomes, harmful or iatrogenic effects, and side effects (Lilienfeld, 2007; Dimidjian and Hollon, 2010; Linden, 2013; Parry et al., 2016). Inadequate treatment choice, the patient’s particular mental conditions, or the therapist’s technical mistakes are typical variables related to unsuccessful and negative outcomes. In recent years, there has also been an increased awareness in the field that the therapists’ individual skills make a significant contribution to the variance in outcome (Baldwin and Imel, 2013; Owen et al., 2015; Hill et al., 2017). While some therapists are generally more successful than others, most therapists have experienced both therapeutic success and failure in different cases (Okiishi et al., 2003; Wampold and Brown, 2005; Kraus et al., 2011; Baldwin and Imel, 2013). However, therapists often have difficulties in identifying their own shortcomings and are unfamiliar with the methods and criteria for identifying and preventing negative outcomes (Dimidjian and Hollon, 2010; Gold and Stricker, 2011; Hilsenroth et al., 2012; Kächele and Schachter, 2014). Accordingly, we need to learn more about within-therapist differences in order to understand what makes even well-trained psychotherapists fail in some cases (Merten and Krause, 2003; Baldwin and Imel, 2013). Recognition of treatment failures is a characteristic of good therapists and may significantly improve clinical outcomes (Hatfield et al., 2010; Linden, 2013; Budge, 2016).

To explore and test putative mechanisms of unsuccessful psychotherapies, we need both quantitative assessments and individual idiographic approaches (Barlow, 2010). These were attempts made early in the history of psychotherapy research. Bent et al. (1976) studied correlates of successful and unsuccessful psychotherapy and found that patients who were satisfied with therapy described their therapists as warmer, more likable, active, and involved than those who were less satisfied. Strupp’s systematic comparison of contrasting cases demonstrated that therapeutic success was connected to the patient’s ability to take advantage of the therapist’s particular relational stance. He also found that the therapist might be able to adapt the relational style to the needs of some patients, but not others (Strupp, 1980a). In successful treatment, the patient could form a productive working relationship early in the therapy, whereas the patient’s deep-seated characterological barriers gave rise to insurmountable barriers in the unsuccessful treatment (Strupp, 1980b). The therapeutic outcome was a function of the patient’s character pathology in interaction with the therapist’s ability to manage his or her own countertransference reactions (Strupp, 1980c).

Nowadays, decades later, research is slowly returning to the issue of contrasting outcomes, mostly confirming the early researchers’ conclusions. Comparing a good and poor outcome case of psychoanalysis, Gazzillo et al. (2014) found striking differences in their therapeutic processes. In good outcome case, the patient could disclose and reflect about her experience of the therapeutic relationship. Her analyst was more oriented toward relatedness and could make active use of adequate interventions (such as clarifications and interpretations of conflicts, defenses, and transference). In poor outcome case, the analyst was not able to deepen the patient’s understanding of her psychic life, and the analyst’s interventions were general and not clearly enunciated. Hayes et al. (2015) found that countertransference reactions were evoked, in successful and unsuccessful cases alike, when therapists’ unresolved personal and professional issues were activated by their perceptions of patient characteristics and behaviors. However, in successful, but not in unsuccessful cases, the therapists’ countertransference management gave them new understanding of what was going on in therapy and allowed them to adjust their work to their patients’ predicament. Accordingly, Schattner et al. (2017) compared two contrasting cases, and found, in the less successful case a clash between the patient’s and the therapist’s relational patterns, negatively impacting each of them. In more successful case, such hindrances were made explicit and negotiated, and the therapist could adapt in a flexible way to the patient’s relational difficulties. Hjeltnes et al. (2018) compared young adult patients with the highest and lowest symptomatic changes after taking part in a mindfulness-based stress reduction program, confirming the importance of the match between the participants’ preferences and needs and the treatment modality. The improved participants found the program to be helpful in moving toward an active stance of personal agency, whereas the less-improved participants had difficulties in understanding the treatment principles, which hindered them from finding new ways of dealing with their problems.

To conclude, regarding the psychotherapy process as a multifarious interaction involving the patient, therapist, and the specific therapy method can help us understand what can lead to improvement, stalemate, or deterioration. This might include such factors as the dynamics of the therapeutic relationship, the working alliance, rupture, and repair of collaboration (Safran et al., 2014); as well as the patient-therapist match and both participants’ capacity to form a satisfying relationship (Zilcha-Mano, 2017). A more extensive and systematic review of relevant literature is beyond the scope of this discovery-oriented study.

Prompted by these issues, the present study aimed to examine why therapists were successful in some cases, whereas some of their other patients remained non-improved. How did the patients and their therapists make sense of and reflect on their therapy experiences in good outcome and poor outcome cases? Were there any distinctive features experienced by the participants at the outset of treatment? To explore these issues, we applied a mixed-method design.

While the definition of “successful” and “unsuccessful” outcomes in psychotherapy may vary depending on the specific research questions, study design, and the perspective of the researcher (Ogles, 2013), it may be argued that such outcomes should involve a significant reduction in patients’ self-reported distress levels (e.g., Goodyear et al., 2017). Therefore, we started by selecting contrasting cases, obtained from the same therapists, following the criterion of reliable and clinically significant symptom reduction or non-improvement at termination, thus controlling for the therapist effects. Next, we analyzed patient and therapist interviews concerning their experiences of psychotherapy, and we compared successful and less successful cases within each therapists’ caseload and in toto. Knowing the outcomes at termination, the baseline interviews enabled us to investigate whether any particular differences were already observable early in the treatment.

## Materials and Methods

### Setting

The present study uses archival data from the naturalistic, prospective Young Adults Psychotherapy Project (YAPP). Of the total of 134 patients (73% female; mean age = 22; range = 18–25; *SD* = 2.2), 92 were offered individual psychotherapy and 42 were offered group therapy at the former Institute of Psychotherapy, at that time a specialist unit within the publicly financed psychiatric care services in Stockholm County, Sweden. The patients reported low self-esteem (97%), conflicts in close relationships (66%), depressed mood (66%), and anxiety (55%) (Wiman and Werbart, 2002). Moreover, about one-third of the patients had personality disorders according to the *DSM-IV and ICD-10 Personality Questionnaire* (DIP-Q; Ottosson et al., 1998).

The open-ended psychotherapies in YAPP were aimed at overcoming developmental arrest and improving the patient’s adaptive capacity. The mean duration of individual psychotherapies was 22.3 months (*SD* = 17.2; *Mdn* = 20; range = 0–85) with a frequency of one or two sessions per week. The non-manualized treatments were conducted by 34 psychoanalytically oriented therapists who met weekly in clinical teams to discuss clinical experiences and treatment problems. Treatment outcomes were studied at termination, after 1.5 years, and at a three-year follow-up (Philips et al., 2006; Lindgren et al., 2010).

### Categorization of Outcomes and Inclusion of Cases

Trying to keep the therapist factor constant, we selected contrasting cases from the caseloads of three therapists. As we wanted to explore the experiences of the most improved and least improved patients and their therapists, we followed the procedure of extreme or deviant case sampling (outlier strategy; Teddlie and Yu, 2007). The categorization of outcomes was based on the Global Severity Index (GSI) of the Symptom Checklist-90-R (Derogatis, 1994). To be regarded as a “successful case,” the patient had to belong to the clinical range at baseline and to the functional distribution at termination. Moreover, the improvement had to be statistically reliable, according to Jacobson and Truax’s (1991) criteria. We defined “less successful cases” as patients in the clinical range at baseline who lacked reliable improvement or were reliably deteriorated at termination. As the distribution of the clinical and the functional population overlapped, we calculated the cut-off (0.90) following the criterion “c” and comparing the pretreatment YAPP sample to Swedish norms.

Reliable change (RC) was achieved if the reliable change index (RCI; based on the difference between two time points divided by the standard error of difference) was equal to or larger than 1.96 (*p* < 0.05). For clinically significant improvement (CI), the patients had to achieve both RC and move out of the clinical distribution into the functional distribution. RCI above 1.96 was regarded as deterioration. Seventy patients (80.5%) belonged to the clinical range at baseline; 29 of them showed CI and two RC only at termination, while 20 patients had no RC and three had deteriorated (missing outcome data in 16 cases).

**Figure 1:**
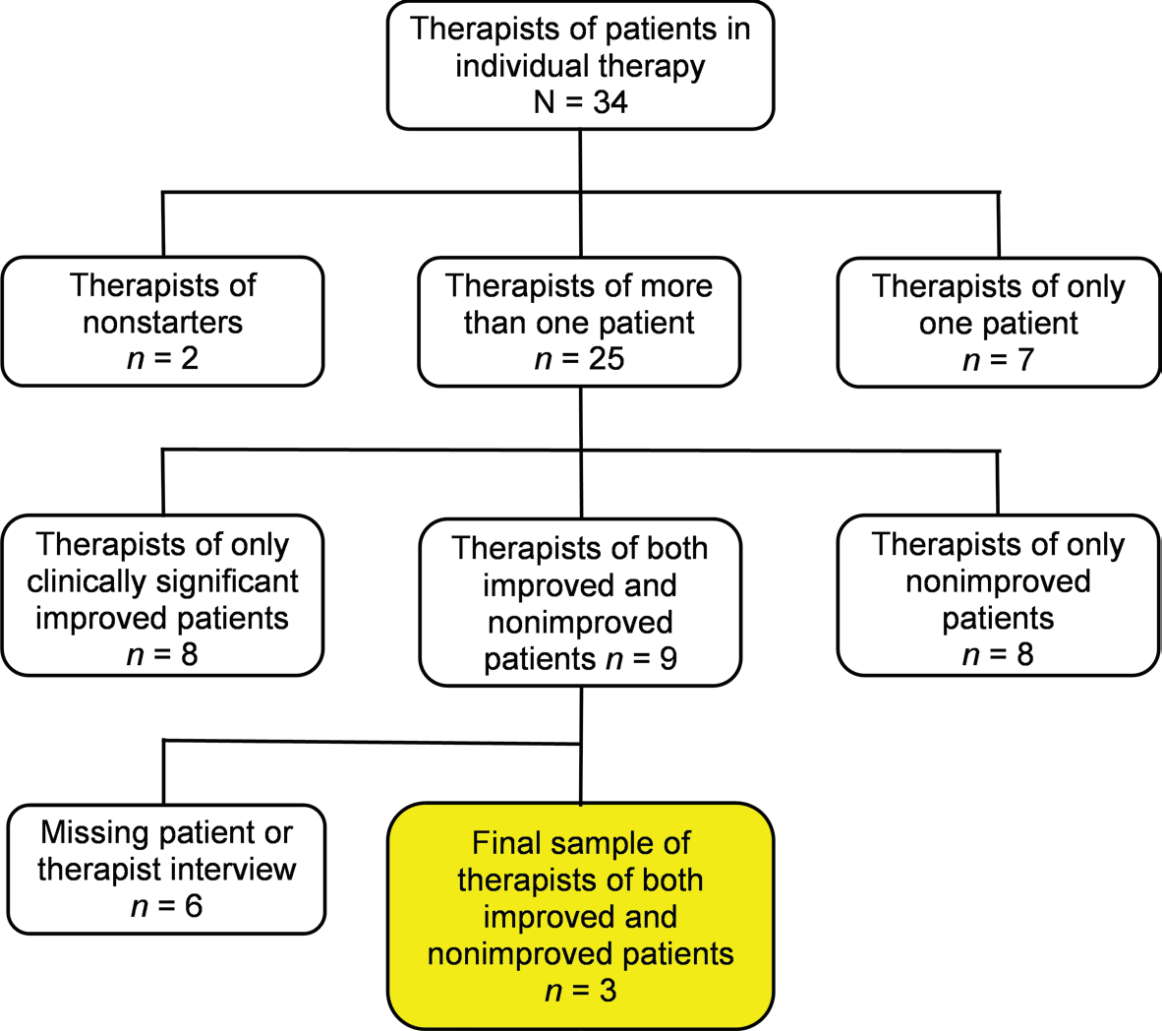
Flow chart from the initial sample of therapists in YAPP to the final sample of therapists of both clinically significant improved and non-improved patients.

Of the 34 therapists in YAPP, two had only patients who never started therapy after the initial contact, seven therapists had one patient each, and 25 therapists had more than one patient (range = 2–7; *Mdn* = 3). In the latter group, eight therapists had only patients with clinically significant improvement, a further eight had only non-improved patients, whereas nine therapists had both clinically significant improved and non-improved patients. In six cases, some of the patient or therapist interviews were missing. Thus, three therapists with two patients each could be included in the present study (Figure 1). One of these therapists had one further patient with CI and one with reliable deterioration; another therapist had one further patient with CI and three patients with no reliable change. In these two cases, we selected the treatments with the largest difference in outcome.

### Participants

The three highly experienced therapists (called A, B, and C) had between 9 and 13 years of experience after being licensed. There were two social workers and one psychiatrist; two were female and one male, aged 50–60 years. Their respective patients have been given names with the corresponding initial letters, ending “y” indicating clinically significant improvement and “n” indicating non-improvement. All the six patients were female and between 18 and 25 years old at baseline. Their axis I DSM-IV-TR diagnoses (American Psychiatric Association, 2000) were major depressive disorder and dysthymia, and their axis II diagnoses were borderline, avoidant, depressive, and not other specified personality disorders. The three patients with clinically significant improvement, but not the three non-improved patients had previous psychotherapy experience.

### Interviews

All patients and their therapists in YAPP were interviewed at baseline (shortly after the initial consultative sessions) and at termination (close to the last therapy session). Thus, the present study is based on 24 interviews. The semi-structured *Private Theories Interview* (PTI; Werbart and Levander, 2006) is aimed at collecting narratives, concrete examples, and illustrative episodes concerning the patient’s complaints and their background, ideas of cure, descriptions of changes, and what fostered or hindered improvement. This interview technique is designed to elicit the informant’s own, open-minded thinking and reduce the influence of the interviewer’s construction of meaning. Furthermore, two questions from the *Object Relations Inventory* (ORI; Diamond et al., 1990; Huprich et al., 2016) were included: “Please give a description of yourself” and “of your therapist” (patient interviews), and “Please give a description of your patient” and “of yourself as just that particular patient’s therapist” (therapist interviews). Upon the spontaneous response, the interviewer encouraged elaboration on each adjective or descriptive phrase, for example, “You said *confused*?” The patients were interviewed by trained clinicians and the therapists by researchers. The audio-recorded interviews lasted about 60 min.

### Qualitative Analysis

Based on the verbatim interview transcripts, we conducted systematic case studies, applying the multiple-case comparison method (McLeod and Elliott, 2011; Yin, 2018) and inductive, experiential thematic analysis (Braun and Clarke, 2006). The case-series methodology enables in-depth examination of cases within their real-life context and of similarities and differences between cases. Experiential thematic analysis is concerned with how people experience and make sense of their life world. Our step-by-step procedure was inductive, as it was grounded in the data and not shaped by pre-existing hypotheses or theories. Moreover, it was explanatory as it involved the researchers’ interpretative activity.

1. The interview transcripts were read line by line. After a first perusal, all relevant sections and paragraphs in each interview were sorted into relevant thematic domains, corresponding to our research questions: the participants’ view of early treatment (inclusive of the patient’s problems and initial ideas of cure), experienced outcomes, retrospective view of psychotherapy, and of the therapeutic relationship.
2. Each interview transcript was coded separately. Similar statements within each domain were clustered into “tailor-made” condensates of central themes that were formulated to be closely related to the participant’s own wording, without interpretation.
3. These condensates were elaborated into narrative accounts, outlining the meanings inherent in each participant’s experience. Here, the thematic domains were explained and nuanced, exemplified by verbatim quotations from the interview transcripts.
4. The contrasting cases of each therapist were compared with each other within each thematic domain. The similarities and differences between the patient’s and the therapist’s narratives were scrutinized.
5. Finally, we compared the three successful and the three less successful psychotherapies.

The analysis was carried out independently by the second and third authors as a part of their master thesis for a five-year psychology program. In the three first stages of data analysis, the coders were blind to the outcomes of psychotherapy. The narrative accounts and comparisons were audited and revised by the first author, a male psychoanalyst and senior psychotherapy researcher. The authors discussed differences in opinions in relation to the original textual data until consensus was reached.

## Results

We start by presenting the three therapists’ contrasting cases from the viewpoint of the patient and the therapist. We then move on to compare each therapist’s successful and less successful case. Finally, we look at what was common for the three successful and the three less successful treatments.

### Therapist A

A’s patient Ally showed clinically significant symptom reduction after ca. 4 years of therapy once or twice a week, whereas Ann was non-improved at the termination of her less than 1 year’s once weekly therapy.

#### Ally’s View of Psychotherapy With A

At baseline, Ally said that she was depressed and pondered immensely. She had knocked about a lot, which she thought was hard—when her circumstances had become a safe spot in her life, she had to change them. She thought it might help to talk about and understand more of the relationship with her dad, but she was worried about not getting help, as her previous treatment did not help.

At termination, Ally described feeling much better, and she relied on her ability to resolve her remaining problems. She was very satisfied with the therapy and felt great confidence in A, even if ending the therapy was tough. It was helpful that the therapy went on for a long time that she dared to open up and that A had always been there. A became an important guide who helped Ally to think differently, and they could laugh together. Sometimes A could be distant, but Ally thought that the therapeutic relationship by its nature includes distance.

… at first I felt so astonished at her being so quiet. Was it only me who had to talk? I did not know how it was to be in therapy. I thought … it’s different for different people, but I had expected her to talk more. But I had to get used to this, and afterwards or after a while I was content with this. (Termination)

#### A’s View of Psychotherapy With Ally

A said at baseline that Ally had an unsettled life and felt lost, and she hoped that therapy could provide a firm ground for Ally. A was unsure of how Ally’s problems manifested themselves in her everyday life, and A was unsure of Ally’s endurance and expectations for psychotherapy. They had talked about whether Ally was prepared for regular sessions and how therapy works. Ally expected A to be more active, but she said that it might be useful in the way A described therapy. From the outset, they started to work with Ally’s maternal transference and A was pleased with Ally being so involved in that work. A described herself as focused on understanding, listening, not being too motherly, and partially holding back her concern, as Ally was on the point of freeing herself from her mother.

At termination, A said that she felt a strong interest in and really liked Ally, which she believed contributed to the improvement. She allowed herself to feel maternal affection, and it was very exciting and rewarding to work with Ally. Initially, A did not understand the extent of Ally’s problem and it was difficult to establish a bond. Ally had a tough time, as she was unfamiliar with the situation. After half a year in therapy, they had a crisis, as Ally thought it was hard to focus so much on her problems, and it was a real eye-opener for A. A began to ask for positive memories, and that became a turning point in their relationship.

I thought I changed, becoming more active. I thought I had been active right from the beginning trying to get her started a little more, her own thinking, etc. But I became more supportive and encouraging after this crisis following my—as I also felt—a rather insensitive intervention, which she experienced as criticism. (Termination)

#### Ann’s View of Psychotherapy With A

At baseline, Ann said that she made high demands of herself and had difficulty feeling she was good enough, especially with boys. Rationally, she could understand that she was just as good as anyone else, and she believed that others perceived her as happy and confident. One of her problems was pondering too much. Perhaps it might help to talk to someone, but it was the pondering she had to change. She knew it would help if she did not make such heavy demands on herself, and it also could help a little to meet a boy.

At termination, Ann said that her problems remained, even if therapy had been a bit helpful. She did not want to be critical, but it was not the right therapy for her, and she did not know if it was due to the method or to A. She knew she had to address her problems but she did not get that help in therapy.

… but I have pondered so much on my problems and my relationship with dad and men and myself so I would gladly accept some more advice on how to think. (Termination)

It was hard that A was so silent, and Ann felt that it was entirely up to her to bring the conversation forward. She got the feeling that A did not know what Ann’s goals in therapy were and she was also doubtful whether A had any of her own goals. Ann perceived A as kind-hearted, but a little meek, awkward, and unsure.

#### A’s View of Psychotherapy With Ann

At baseline, A described jealousy as Ann’s core problem and she wondered if there was something Oedipal in her relationship with her dad. A believed it could be helpful for Ann to talk over her problems.

… she is very reflective herself, this girl, but I think she needs someone listening to her, someone mirroring this, an adult not involved in her sphere. (Baseline)

A perceived Ann as well motivated but having unrealistic ideas of therapy. They had talked a lot about this, including how much support she needed and how fast it would work. Intellectualization was a possible obstacle, as Ann herself said she pondered a lot, but her pondering was filled with emotions, thus indicating an opening. As Ann’s therapist, A described herself as listening, understanding, and adopting a wait-and-see policy.

At termination, A reported that Ann had chosen to prematurely end the therapy. One contributing factor may have been that Ann’s pain and motivation decreased when she met a new boyfriend. Furthermore, it may have been important that Ann wanted more advice, feedback, and quick results and was considering cognitive therapy. A had never given direct advice, but she tried to find a balance between giving and playing back to Ann to make her think herself. Ann missed a good number of sessions, which made it difficult to deepen their work. Her low self-esteem also emerged in their relationship, but they could not talk about it. As Ann’s therapist, A listened a lot with a keen ear and tried to think about the transference. In future, there would be a risk of Ann feeling bad again, but A believed in Ann’s capabilities.

#### Comparison of A’s Two Cases

##### Early in Treatment

Ally and A had more convergent views of her problems and of what could be helpful, thus facilitating their subsequent work. Ann expressly wanted more than talking, as she thought her pondering was an obstacle, whereas A believed that talking over Ann’s jealousy would be helpful. In both cases, A stressed the importance of listening, mirroring, and firm therapeutic boundaries. However, in case of Ally, A was keen on not being too motherly and concerned, whereas in case of Ann, A wanted to be cautious and maintain a wait-and-see attitude. A described early in-session enactments of Ally’s problems and Ally’s active contributions to the resolution. They talked about their different ideas about A’s activity in treatment, and they were able to come to an agreement. Even Ann and A had talked about their divergent ideas of therapy; however, neither of them mentioned what their talking led to. Furthermore, A described more feelings and stronger initial involvement in the case of Ally than with Ann.

##### Experienced Outcomes

At termination, Ally and A had a more convergent view of changes and described the treatment as successful, whereas both Ann and A described the treatment as unsuccessful. Ally was very satisfied with her therapy and was confident in doing well on her own, whereas Ann felt that her core problems remained and she wanted to find an alternative way. A believed in Ally’s ability to cope with future stress. In case of Ann, A’s view of the outcome was vaguer and more contradictory; she noticed limitations in their work and was hesitant about whether Ann could deal with her remaining problems but believed in her resources.

##### Retrospective Views of Psychotherapy

Also, the views of what was going on in therapy were more similar in the case of Ally and A. A common theme in both patients’ narratives was A’s degree of activity and silence, but they experienced it in different ways. Ally felt that she profited a lot from their work, even though she initially wondered about A’s silence and even though ending therapy was tough. Ann was dissatisfied with the therapeutic approach and with A; too much was left to Ann. A experienced her work with Ally as exciting and rewarding, whereas in case of Ann, it was difficult to deepen the contact. A gave several concrete examples of her work with Ally, whereas she focused on what was impossible to work through with Ann. Productive work with Ally’s maternal transference could start early on, whereas A’s thoughts about Ann’s (paternal) transference did not seem to result in any joint exploration. A significant turning point in therapy with Ally was a crisis in their collaboration and its resolution, when A gained a new understanding of how Ally experienced her interventions and adjusted her technique accordingly. Furthermore, A described how her view of Ally evolved throughout the treatment, whereas she did not mention such developments in case of Ann. A knew that Ann wanted more advice and feedback, but she did not reflect in her interview on confronting Ann with their incompatible views or adjusting her approach. Instead of ruptures and resolutions, Ann missed several sessions and initiated premature termination.

##### Therapeutic Relationship

Ally felt great confidence in A; A cared for her, and there was space for humor. They also seemed to have done some work on difficulties in separating and in ending therapy. Even though Ann mentioned that A probably cared for her, she described A in negative terms and she emphasized their poor match. A described her maternal feelings, personal involvement, and own gains in her relationship with Ally, whereas her relationship with Ann was more distanced and marked by insecurity.

### Therapist B

B’s patient Bonny showed clinically significant symptom reduction at termination of her more than two and half year’s twice weekly therapy, whereas Brynn was reliably deteriorated in terms of symptom severity after 4 years in twice weekly therapy.

#### Bonny’s View of Psychotherapy With B

Bonny described at baseline that she was depressed, on sick leave and taking antidepressant medication. She was ashamed of her parents, and she believed that their big problems had given rise to hers. Consequently, she never allowed herself to have a boyfriend, never let others get close to her, and she felt incredibly lonely. She wanted to regain her self-esteem, to work on her relationship with her parents, and to move on. She was aware that she had barriers hindering her from really telling everything in therapy.

I am just so afraid of not getting any help. Because I didn’t get it earlier in my life … it feels like connected with the fact that I never felt understood, like it does not matter how many times I am sitting here and telling things about myself, because it feels like no one can understand how I feel it anyway. (Baseline)

At termination, Bonny felt much better and did not have any problems. She believed both the therapy and her everyday life contributed to improvements. She was satisfied with her therapy; it was good to talk over her thoughts with B. Nevertheless, she wished B was more like a mother and gave her advice. Bonny had confidence in B and that B cared for her; often she felt relieved after sessions. However, it was difficult to speak out and maybe she had not done it yet. Bonny described B as considerate, understanding and helpful; they could have fun together.

#### B’s View of Psychotherapy With Bonny

B thought at baseline it was strange that Bonny had done so well despite her parents’ major shortcomings. Now she had broken down, and B was very worried about her. B hoped therapy could be a safe place and help Bonny get to her feet again. Perhaps she overestimated this prospect and was uncertain if Bonny would continue in therapy. Bonny had a very negative view of adults; she was hostile and suspicious and tried to manipulate B to abandon her therapist role.

… when I presented her case to our team, I could understand that she, in a way, recreated with me a climate where it was very difficult to feel empathy for her, to feel commitment. So she wants help and at the same time she is counteracting it. (Baseline)

This understanding helped B, and thenceforth a challenge was to create a confiding relationship. B had to be extra careful and educative about therapy, about therapeutic boundaries, and what she could expect from Bonny. She could understand that Bonny wanted to know who B was and if she could help her. Bonny’s reaction to B’s recently cancelled session was a good sign, as it showed she could express her anger. B thought she had to be patient and endure challenges, but she was also impressed by Bonny being so open with her fantasies.

At termination, B believed it had been helpful for Bonny to meet a sensible adult who allowed Bonny’s needs to guide their work, even though Bonny also had to wrestle with B being an adult. Bonny progressed from having a lot of contempt to being increasingly open with B. B referred to several helpful interpretations, for example, when she addressed Bonny’s distrust. It was helpful not only to be explicit with the boundaries but also to be flexible when required, and to develop a close, trusting mutual relationship, where Bonny could fill up the gaps and go in search. Love in the therapeutic relationship was also important, as well as shared humor.

#### Brynn’s View of Psychotherapy With B

At baseline, Brynn complained that she had lost her curiosity. She was stuck in her thoughts, had difficulty focusing, and thought she behaved badly and nastily. She had been going downhill for a long time, acting in a way that did her harm. Her relationship with her family was complicated, and she blamed them for her problems; they helped her too much, and she became incapable. Her relationship with her boyfriend was in a muddle; both were unfaithful, and Brynn did not know why she was with him. She has had sex with many guys, even those she did not want, and she felt it had ruined her. Brynn wanted help to remove focus from herself and from thinking so much. She wanted to cleanse herself to be able to move on.

At termination, Brynn said that her problems remained, and she was feeling worse. She believed therapy had contributed to the deterioration, and she did not agree with B that it was a pity to stop. She did not trust B because of things she said.

She also said once that I am a whore, but she said she did not, so I told her I must be very seriously ill if I hear voices… and she said it does not belong to her vocabulary, possibly “promiscuous.” And the second time, she was just quiet, but when I picked this up again three weeks later she said she had not said that either. I mean I could not get these two great things all wrong. (Termination)

B was distant, and Brynn wondered if B really cared for her. Sometimes Brynn saw emptiness in B’s eyes, and there was no closeness. She had repeatedly claimed she needed more support, but it was like B did not understand. Brynn wanted more of a dialogue, more structure in their conversations, as she often talked about unnecessary things. She was stuck in old patterns and wanted help to move ahead. Brynn did not want to blame B, but she believed B had her own problems.

#### B’s View of Psychotherapy With Brynn

B described at baseline that Brynn avoided taking responsibility and laid the blame for her problems outside herself. Her parents had failed in their responsibility and allowed Brynn to play around, without providing a “holding environment” and without setting limits. B saw Brynn as both strong and at a breaking point; she was worried that Brynn was in big trouble. Brynn needed help daring to trust others and to see that she had value. B wanted to be the one that Brynn had missed, “holding” Brynn and at the same time setting limits, which would be difficult. There was a connection very early between them, although it was uncertain whether Brynn trusted B. As Brynn’s therapist, B described herself as curious and moved.

At termination, B experienced Brynn’s decision to end the therapy as an unfortunate tragedy, because they had just started to come closer to each other. Brynn acted as in other relationships, she destroyed. B also wondered if her sick leaves had made Brynn worried that B would leave for good, so that Brynn felt forced to break up. They had done a good preparatory job, and it would be good if Brynn could resume therapy. Initially, B had difficulty getting space to say something, but it became more of a dialogue and closeness developed.

Our relationship changed over time in a noticeable way, toward an increased closeness. But when it became too close I could see how she actually turned against me by saying that she had started to analyze me and she claimed “how can you say that I’m a whore,” for example. I mean there were such strains of paranoia, it sneaked into the room and was impossible to deal with; it could not be interpreted or talked about, and this was escalating. (Termination)

It was sad that Brynn felt such distrust, but B thought they still had a sustainable and loving relationship. B believed Brynn wanted more support and advice than she received. B found it hard to tell about this therapy; the sessions had often been fragmented and confusing. B had to fight; this therapy required both immense presence and containing. Brynn was the one who had affected her most in 30 years.

#### Comparison of B’s Two Cases

##### Early in Treatment

In both therapeutic dyads, the participants described the patient’s life circumstances in a similar way and the therapist expressed her great concern. However, B elaborated and provided a contextualized conceptualization of Bonny’s problems, whereas she repeatedly questioned Brynn’s view and interpreted what Brynn told her in a different way. As to their initial ideas of cure, Bonny and B were more in accord. Bonny expressed her hope that the therapy might help her, and B had clear ideas of her stance working with Bonny—it would be necessary to work in a way she usually did not and to adjust her approach to Bonny. By contrast, B’s ideas were more general in the case of Brynn—she wanted to compensate Brynn for parental failures rather than adjusting herself to the patient. B did not mention Brynn’s most important goal, getting help in being less self-focused and in pondering less, and to cleanse herself. B described an early and loaded situation when she and Bonny had to talk about what Bonny could expect in therapy, and she expressed her understanding of Bonny’s emotional reactions in sessions. In case of Brynn, B thought it would be difficult to give her what she lacked. B expressed both her insecurity and an awareness of challenges in the work with Bonny, whereas she seemed to be more confident in the case of Brynn, without being specific about her tasks.

##### Experienced Outcomes

At termination, both Bonny and B described the treatment as successful. Brynn described her therapy as a failure, whereas B thought they had just started fruitful work. B was more confident of positive changes and what contributed to them in the case of Bonny, whereas her picture of Brynn’s outcome was more inconsistent—it was good preparatory work, which B described in a similar way as in the baseline interview.

##### Retrospective Views of Psychotherapy

Bonny and B had convergent views of their joint work, whereas Brynn’s and B’s views were incompatible. Bonny presented a positive picture of her therapy and B, although she also mentioned what she had lacked. Brynn, on the other hand, was upset talking about her therapy, giving many examples of what gave rise to her dissatisfaction and what she would like to have instead. B described how she and Bonny could work at overcoming obstacles and how her interpretations could be helpful, whereas this was impossible with Brynn—B felt overwhelmed with things that just happened.

##### Therapeutic Relationship

Likewise, the pictures of the therapeutic relationship were similar in the case of Bonny and contradictory in the case of Brynn. Both Bonny and B described their deep relationship with for a shared sense of humor. Brynn experienced distance and emptiness, instead of the closeness and mutuality described by B. With both patients, B mentioned love in the therapeutic relationship; however, there was a difference in how involved B was with her patients—Brynn was the one who had affected her most in her career.

### Therapist C

C’s patient Cindy showed clinically significant symptom reduction at termination of her 19 one and half year’s once-a-week therapy, whereas Caitlin remained unchanged in terms of symptom severity after less than 2 years in once or twice weekly therapy.

#### Cindy’s View of Psychotherapy With C

Cindy said at baseline that she felt depressed, unsure, and without a consistent identity. Instead, she was putting up a harsh façade and setting high goals. She could not let anybody get closer to her, as she knew that losses hurt. Cindy thought she tried to be perfect to gain control of the situation in her childhood, when both her parents were sick and her father died. However, she had difficulty remembering her childhood, which she thought was a defense mechanism. She had been in therapy before, which did not help, and now she had to try risking failure. She wanted help to feel normal, to gain better self-esteem, to be able to maintain close relationships, and to find less demanding things to do. A positive change started prior to therapy when her boyfriend found a way to get close to her, as no one had been before.

At termination, much had improved, and the problems were small. It was helpful to talk and think about certain things, which gave understanding and insight into what she wanted. Occasionally, she felt worse, and the silence was tough, but she could get on with things without being forced.

We could sit silently for fifteen minutes maybe, because I refused to start talking, but C did not start either. Then he could say just *hmm*, and I said *hmm*, and then we waited for me to think of something, because sometimes it felt like my head was completely empty. But he was convinced that in psychotherapy you should talk yourself … I had to associate freely; he was very stubborn, not leading me in any direction. (Termination)

Much in her life became more stable, which also helped her feel better. Cindy experienced C as patient, persistent, and helpful, not controlling the conversation. Cindy was afraid of deterioration and of not being able to get along without therapy, but hopefully, it would go well.

#### C’s View of Psychotherapy With Cindy

At baseline, C described Cindy’s background as traumatic. In addition to the parents’ illnesses, he also mentioned other possible traumas, but Cindy did not pick up on this. In therapy, Cindy needed to get in touch with her feelings. She was already on her way, feeling more pleasure in things and less pressure. She was likable, keen, and could put her foot down. She used strong defenses, such as intellectualization, and silence in therapy made her unsure. As Cindy’s therapist, C was understanding, empathetic, and committed.

At termination, C thought that talking about traumas that eventually came up was most helpful. Cindy seemed to deteriorate for a while and was critical and lacking confidence for a long time, but this changed. C had to resist Cindy’s vehement attacks on him and the therapy. A turning point was when he realized how unhappy she was, and prolonged therapy. This, along with him not flinching from talking about trauma, fostered her confidence.

When she talked about what she was exposed to and then did not want to talk more about it, anyhow, I forced her to come back by saying that this is important, you need to talk about this if anything is to happen, if there is any meaning to this. (Termination)

When Cindy was offended by something he said he could handle it. He had not only interpreted psychoanalytically but also used positive reframing, which had a good effect. At termination, Cindy remained skeptical of the method but still satisfied. The improvement seemed to be lasting, and Cindy had great potential to cope with new stresses.

#### Caitlin’s View of Psychotherapy With C

At baseline, Caitlin talked about a turbulent relationship with a woman, and she thought she lacked a clear sexual identity. She was unsure; she adapted to others and put them on a pedestal. Another problem was that she easily got embarrassed, had trouble meeting people, and shut herself up. She easily became absorbed by problems instead of dealing with them. She had few childhood memories, and there were things she did not dare to think about. In therapy, Caitlin wanted to reclaim and understand herself better.

At termination, many of her problems remained unchanged, but there were some improvements. She got help to discover her repetitive patterns, turn negative perspectives into positive ones, and mourn her relationship with the woman. It was helpful to have had someone by her side; she could come out with her opinions and sorrows, even though she did not tell everything.

There are nuances in me that I find hard to express because they feel ridiculous and I am very uncomfortable with them, and I did not succeed, could not even manage to talk about them in therapy. Sometimes I think I did not reach out because I did not convey the whole feeling. (Termination)

Ending therapy was hard at first because she felt nothing had happened, but later on, she took the view that she could talk in therapy without making changes and would be able to continue on her own with her new tools. Caitlin thought that the improvement was partly due to the passage of time and that she might improve even without therapy. Sometimes she felt that the therapy was disturbing rather than helpful, and she became more self-focused than she wanted. She described C as calm, confident, amusing, and perspicacious; he made her feel seen, but owing to her fear of conflict, she could not say anything negative about him. The sessions were never tough, but on occasion, she had been angry at him without expressing it, for example, when he asked about change, whereas she wanted to grumble.

#### C’s View of Psychotherapy With Caitlin

At baseline, C said that he knew only a little about Caitlin’s background and nothing about any trauma, but he had an idea that her parents influenced her identity development. Caitlin had difficulty showing anger, and she would be helped by acting out her feelings and finding her identity. Outside therapy, she had to sort out her relationship with the woman and finish her studies. Caitlin was nice, and C wondered if she idealized and tried to please him. She talked a lot and sometimes needed to be stopped. C said it was difficult to describe himself as Caitlin’s therapist, but he tried to listen, understand, and confront her in a sympathetic way.

At termination, C said they prolonged the therapy by 1 year and he thought she still needed more therapy. However, without a time limit she would keep harping on the same theme.

She could talk for 45 minutes without stopping, and I would wonder how much feeling was there behind it, and this changed during the course of therapy, so you can say there was a certain obstacle, her intellectual defense. (Termination)

It was difficult to understand her problems, as she never mentioned any trauma. Working through the termination gave Caitlin tools, although she was afraid of not being able to make choices without therapy. Caitlin had been helped by making positive changes outside therapy. C’s countertransference was impatience when nothing happened. Rather than making interpretations, he was supportive but also confronting when she said something contradictory, difficult to understand, or did something self-destructive.

#### Comparison of C’s Two Cases

##### Early in Treatment

Both Cindy and Caitlin mentioned difficulties in remembering childhood. Cindy thought this could be a defense, whereas Caitlin did not understand why it happened. C noticed traumatic experiences in Cindy’s background and not being able to see any traumas in Caitlin’s background. Both Cindy and C mentioned concrete changes that Cindy should make. Caitlin’s view of the therapeutic goals was more diffuse, whereas C had a definite view of what she needed to change in her life. Cindy talked about a change process that had already started before therapy, whereas Caitlin described her increasing problems. C was hopeful about Cindy’s therapy but wondered how Caitlin’s would go. He felt that Cindy could stand up to him but suspected Caitlin of being compliant and idealizing him. He experienced himself as confronting Cindy but found it difficult to describe his way of working with Caitlin.

##### Experienced Outcomes

At termination, both Cindy and C described Cindy’s positive changes, whereas Caitlin and C experienced Caitlin as mostly unchanged, even though she had some new tools. Both patients linked their improvements to factors outside of therapy, but Cindy stressed that the therapy had contributed. Cindy was afraid of deteriorating without therapy, whereas Caitlin believed in the change process starting after termination. C’s views were the opposite: he thought Cindy needed to end her therapy, whereas Caitlin needed more therapy, as she was afraid of not coping on her own.

##### Retrospective Views of Psychotherapy

Cindy and Caitlin were both skeptical of the therapeutic method. Nevertheless, Cindy felt therapy helped and wondered how to get along, whereas Caitlin was more critical, saying that the therapy did not contribute to change and that it was good to end it. Cindy described the sessions as periodically tough and Caitlin as never tough, but there were things Caitlin could not bring up with C. Both Cindy and C thought it was helpful to prolong the therapy and not to flinch from addressing ticklish subjects. Neither Caitlin nor C described prolonging the therapy as positive and both of them experienced setting a time limit as helpful. C described dealing with Cindy’s criticism and attacks on him and the therapy, whereas with Caitlin he had to deal with her rumination and intellectualization. In both the cases, he deviated from the psychoanalytic method and was more supportive. C emphasized the work on traumatic experiences in the case of Cindy and the lack of it in the therapy with Caitlin.

##### Therapeutic Relationship

Cindy’s view of her relationship with C covered both positive and negative aspects. C could see and appreciate this. Caitlin’s view was clearly positive, but she revealed her fear of conflict, which hindered her from showing anger or saying something negative. C seemed not to be aware of her being skeptical of him and the therapy. In case of Cindy, C described how he worked to gain her confidence and with his negative countertransference. In case of Caitlin, he focused on her avoidance and defenses, but he mentioned that he sometimes felt impatience.

#### Successful Therapies

The comparisons within the three therapists’ contrasting cases revealed that in the successful treatments, the patient and the therapist shared an early common understanding of the presenting problems and what could be helpful. At baseline, the therapists experienced good comprehension of the patient’s difficulties and developed an individualized conceptualization of their problems and background. From the beginning, the therapists presented a clear picture of their ways of being with the particular patient. All the therapists described an early staging of the patient’s problems or a crisis in their relationship, which together they could work through. Both Ally and Bonny were anxious about not getting help, and their therapists referred to their work on the patients’ fears and expectations. In all successful cases, the therapists actively fostered a confident relationship and were personally interested in their patients. The participants shared a view of the therapeutic relationship as both supportive and challenging. The patients experienced their therapists as helpful and considerate. Ally and A, as well as Bonny and B had a good time together; however, Cindy presented a more critical view. In all successful cases, the therapists provided a clear picture of their therapeutic work, giving several specific examples of dealing with obstacles to collaboration and how they worked actively on important aspects of the patient’s difficulties, as these unfolded in sessions. They adjusted their working style to their patients’ needs, deviating from their usual stance or from the method. They presented a positive picture of their patients, of successive developments, and of the deepening of the therapeutic relationship, although this process was not without obstacles. At termination, the patients and their therapists had a convergent view of improvements; they were satisfied with their work and confident with each patient’s future, even though they also expressed some concern about how the patients would deal with new stresses after therapy.

#### Less Successful Therapies

Early in the less successful treatments, the therapists seemed to have missed some important aspects their patients regarded as important parts of their problems, interpreted them differently, or did not acquire an accurate conceptualization of the patient’s problems and their background. Later on, these missing aspects and expected difficulties had an essential influence on the therapeutic process. The participants’ views of what could be helpful were mostly incompatible. The therapists’ picture of the future therapeutic work was indistinctive and formulated in general terms. The therapists described obstacles to the therapeutic collaboration but not their way of dealing with them. They tended to disregard their own role in the interactions and to explain difficulties as a consequence of their patients’ problems. At termination, the patients and their therapists had contradictory views of the therapeutic work and gave diverging descriptions of the outcomes. Both A and B focused on what was not possible to work on or to deepen, and they attributed the hindrances to the patient. They also thought that their patients wished for another approach; however, they did not draw conclusions from this or alter their approach. Ann and Brynn were openly dissatisfied and lacking confidence in their therapists, whereas Caitlin stressed some positive aspects. Both Ann and Brynn decided to end their therapies and to look for other treatments, whereas Caitlin thought she could do better even without therapy and would start the change process after termination. At termination, the patients expressed dissatisfaction with their therapies and experienced that the therapy did not help or contributed to impairments. They wanted to quit the therapy, whereas the therapists thought that their patients needed more therapy.

## Discussion

This study aimed to explore contrasting cases of successful and less successful psychotherapies conducted by three therapists. Comparing the patients’ and the therapists’ accounts of their therapy experiences, we found both differences and similarities, both between the contrasting cases and between the therapists, indicating the uniqueness of the therapeutic interactions and the multitude of factors influencing the therapy process in a complex, synergistic, and mutually reinforcing manner. Nevertheless, the main differences were already manifest at the outset of treatment. Within the constraints of a journal article, we are able to contextualize our results only in relation to the selected choice of relevant research literature.

### Differences Between Successful and Less Successful Therapies

#### Early in Treatment

In the successful cases, the therapists gave an elaborate picture of their patients’ problems and background, consistent with the patients’ presentations. In less successful cases, the therapists misinterpreted or disregarded some aspects that the patient described as important and could present an unclear image. According to Oddli and Halvorsen (2014), experienced therapists are able, early in treatment, to provide contextualized, individualized conceptualizations of their patients’ problems. If a therapist does not pay attention to some core aspects of the patient’s difficulties, as experienced by the patient herself, this may be a major obstacle in the future therapeutic work. Accordingly, Silberschatz (2017) found that if the patient experiences the therapist as sensitive to her problem presentation, she may feel more support and have a more positive view of the therapy, which is linked to a better outcome.

Consequently, in the successful therapies, both parties were more in accord about what would be helpful. From the outset of treatment, the therapists could flexibly adapt their therapeutic stance to their patients’ expectations, needs, and capacities, and in two of these cases (A and B), this involved the therapist’s active attempt to discuss with the patient what to expect in psychotherapy. In this way, the therapists contributed to building a “good enough” sense of collaboration, preventing dropout and creating a “working space,” with room to introduce new ways of addressing the patient’s concerns (Horvath et al., 2011). All the patients in the successful cases overtly expressed their fears, inner barriers, or determination to make an effort. This was not found in the less successful cases, and the therapists seemed unable to establish a sustainable sense of mutual collaboration. A further contribution to effective processes in successful cases was early staging of the patient’s problems, or a crisis in their relationship, followed by repair of collaboration (cf., Safran et al., 2014). This was not reported in the less successful cases.

One reason for the therapist missing important aspects of the patient’s difficulties or ideas of what would be helpful can be the therapist’s strong positive or negative countertransference (Hayes et al., 2015). Many therapists react adversely to a patient’s negativism and hostility. In such cases, the therapists’ ability to curb countertransference reactions and their skills in eroding barriers to human relatedness might play an important role in the outcome (Strupp, 1980b). For example, therapist B described her early strong countertransference feelings with both of her patients. In successful case, reflecting on the patient’s transference and her own countertransference guided her in modulating her stance to suit the patient’s needs, capacities, and expectations. In deteriorated case, B wanted, from the very beginning, to compensate her patient for what she had missed but anticipated difficulties in setting limits. At termination, B described this patient as the one who touched her most of all. This “exceptional” patient seems to have hooked into the therapist’s fears and desires, rendering it difficult for her to take a “third position” (Benjamin, 2009; Bimont and Werbart, 2018). Furthermore, in the successful case, B paid attention early on to potential obstacles and her own hesitation, whereas her expectations were more positive in the unsuccessful case.

In a previous study of non-improved cases, the therapists experienced the therapeutic collaboration, early on, as especially stimulating. They seemed to underestimate their patients’ problems and their unprocessed positive countertransference contributed to the view of being on the right track. At termination, they concluded that the patients needed more time in therapy, attributing the limited progress to the patients’ resistance rather than their own limitations (Werbart et al., 2018). On the other hand, in successful cases, the therapists described active, relational work that included paying attention to incongruities in the patient’s self-presentation and being mindful of the patient’s avoidant behavior. Their early dual focus on both possibilities and hindrances to the therapeutic task seemed to strengthen both the patient’s and the therapist’s motivation (Werbart et al., 2019). Accordingly, Hayes et al. (2015) found *fewer* unpleasant feelings and problematic countertransference reactions expressed in interviews by therapists in unsuccessful cases than by therapists whose outcomes were successful, whereas Oddli and Halvorsen (2014) reported that successful therapists expressed their own uncertainty, especially at the outset of therapy.

#### Experienced Outcomes

In the successful cases, both parties presented similar pictures of positive changes. In less successful cases, all therapists saw more improvements and paid less attention to remaining problems than their patients; however, two of them hoped for post-therapeutic developments. Such myth of improvements initiated by termination could make the therapists blind to failure to progress in treatment. According to Lambert (2011), non-response to treatment seems to be connected with the therapists’ tendency to neglect lack of change and await future improvements, and a failure to take necessary measures. At termination, the non-improved patients were clearly dissatisfied. Two of them thought that they would needed another form of therapy, whereas their therapists thought that they needed more of the same. Thus, lack of early negotiation regarding the patient’s ideas of cure had lasting consequences for the patients. Preparing patients for psychotherapy and negotiating divergent perspectives on treatment goals and tasks can contribute to a stronger working alliance and improved outcome (cf., Horvath et al., 2011; Schattner et al., 2017).

#### Retrospective Views of Psychotherapy

In the successful cases, all the therapists gave a rich picture of their therapeutic work, providing multiple specific examples, whereas in the less successful cases, the descriptions were vague and unspecific. Furthermore, in the successful cases, the therapists described how they adjusted their therapeutic stance to their patients and balanced between giving support and challenging. By contrast, in the less successful cases, the therapists failed to adapt to their patients’ needs. Therapist C thought he was doing it even in the less successful case; however, he disregarded his patient’s need for more challenge and less support. Therapist B seemed to be too challenging in the unsuccessful case, and her therapeutic stance was marked by unresolved countertransference issues; thus, she was unable to keep an optimal balance between professional and personal aspects of involvement (cf., Schröder et al., 2015).

Both in successful and less successful cases, some patients experienced periods of impairment. When working on painful issues, adequate interventions might result in more unstable defenses and increased symptoms. In such periods, the therapist’s task is to help the patient to process the emerging feelings without fearing the patient’s strong reactions, being there for the patient in charged moments (Barber et al., 2013). In less successful cases, the patients did not experience such help. This could be interpreted as indicating the therapists’ difficulties in reflecting on their own contributions to their patients’ failure to improve, taking in negative feedback from their patients, and adjusting their work accordingly.

#### Therapeutic Relationship

In successful cases, the patients presented at termination a more positive picture of the therapist and their relationship. In case C, however, the patient in the successful therapy gave a mixed picture of the therapist, being explicit about negative aspects of the therapeutic relationship, whereas the patient in the less successful therapy was openly positive but hinted at unvoiced negative experiences. The therapists in the successful therapies, but not in the less successful ones, described how they worked with emerging difficulties in the therapeutic collaboration; they monitored the patient’s resistance from the beginning, as well as their own ways of being with the patient. Both parties seemed to contribute to the patient’s secure attachment to the therapist, providing the patients with a secure base for expression and exploration of their painful feelings and thoughts (Mallinckrodt, 2010). In cases A and B, both parties in the successful therapies gave examples of corrective emotional experiences, resulting in the patients finding new ways of relating to others. On the other hand, the patients in the less successful cases experienced a poor match with their therapists. In case B, the patient felt her relationship with the therapist was a repetition of her problematic family relationship, whereas the therapist wanted to be the one the patient missed in her family of origin. The therapeutic relationship in the less successful case of C seems to have been grounded in both parties’ distorted views. The therapist was looking for absent traumas and believed he matched his stance to his patient’s needs. The patient thought she could do as well without therapy. She concealed her negative views, behaving in a compliant way. These cases are clear examples of a clash between the patient’s and the therapist’s relational patterns, a clash that negatively impacts each of them (Schattner et al., 2017). What hindered open statement and negotiation of differences and disagreements seems to have been collusion between the patient’s transference and the therapist’s countertransference.

#### Factors Outside of Therapy

Even though the present study focuses on within-therapy factors, alternative interpretations of the results might take into consideration a broader context of the patients’ life circumstances. Successful therapeutic work could be facilitated by the fact that all the recovered patients had previous disappointing therapy experiences. It is possible that people undertaking a new therapy commit themselves to being open, honest, and vulnerable in ways that enable their therapists to do good work with them (cf., McKenna and Todd, 1997). Accordingly, in our previous studies, the proportion of patients with previous psychotherapy experience was higher in the successful cases than in cases of non-improvement (Werbart et al., 2018, 2019). Furthermore, in the successful cases, the patients mentioned supportive life circumstances and getting support in close relationships (Palmstierna and Werbart, 2013), whereas non-improved patients reported both helpful life conditions and negative impacts of life events (Werbart et al., 2015). Thus, from the patients’ perspective, psychotherapy can be considered as one component in a life-long process of working through of psychological stresses rather than a place for a decisive and complete cure.

### Within- and Between-Therapist Differences

Looking at within-therapist differences, we found that the therapists could function in a highly experienced way (cf., Oddli and Halvorsen, 2014; Hill et al., 2017) in successful, but not in less successful cases. What differed between the contrasting cases was slightly different for each therapist, but substantial differences appeared early in the treatment. For example, therapist A adjusted her way of working more to her patients’ needs in the successful therapy, whereas the ruptures in collaboration (Safran et al., 2014) were not resolved in the less successful case. Therapist B described the therapeutic alliance as stronger, and the parties’ view of the alliance was also more convergent, in the successful case. Therapist C managed to balance between supporting and challenging only with his recovered patient. These differences can be interpreted as due to the quality of the therapeutic relationship (as experienced by both protagonists) rather than to patient psychopathology. The difference between good and poor quality of the therapeutic relationship seems to be due to some aspects of the patient-therapist dynamic match. These aspects might be understood in terms of specific transference-countertransference configurations. Thus, the therapists’ capacity to “mentalize” countertransference seems to be decisive (Barreto and Matos, 2018).

Furthermore, we found marked differences in how consistently the three therapists worked with different patients. It is easy to recognize therapist C’s self-description of his work regardless of which case he was describing. For example, in both cases, he was looking for previous traumas and seemed to adapt the same therapeutic stance. In less successful case, he did not notice how much his patient did not disclose to him. Therapist A seemed to be the most flexible, and there were obvious differences in her two self-descriptions. Therapist B can be placed in a midway position: in the successful case, her therapeutic stance was more suited to her patient’s characteristics, whereas in the deteriorated case, her early countertransference affected her view of the therapeutic goals and tasks. This contradicts the idea of keeping the therapist factor constant. How “constant” the therapist factor is, is itself a therapist factor.

Accordingly, we found differences in how flexibly the therapists could adapt to their patients’ relational patterns. Comparing two contrasting cases treated by the same therapist, Schattner et al. (2017) found that the therapist’s ability to deal with difficulties in the therapeutic relationship was decisive in the development of the therapeutic alliance and influenced the outcome. In case of negative development, the patient’s and the therapist’s relational patterns clashed, whereas in case of positive development, the disagreements and differences were openly negotiated. These two interconnected aspects are congruent with our findings: in the poor outcome cases, the therapists were less able to flexibly adapt to their patients’ relational patterns, whereas in the good outcome cases, they were able to contribute to repair of ruptures in collaboration (Safran et al., 2014).

Zilcha-Mano, (2017) distinguished the patients’ more stable, “trait-like” tendencies to form satisfying relationships from “state-like,” interaction-related changes in the relational patterns, the former enabling treatment to be effective, and the latter making the alliance therapeutic. Accordingly, in our study, the therapists could more successfully adjust to the patients’ “trait-like” relational patterns in the successful than in the less successful cases. We also found between-therapist differences in this respect, from therapist A’s more flexible interpersonal stance through the clash of relational patterns in case B, to therapist C who did not alter his ways of being working with different patients.

Our study confirms Strupp’s (1980b) conclusion that the therapeutic relationship becomes established and fixed very early in treatment and that it influences its course and outcome. In some of Strupp’s contrasting cases, the quality of the therapeutic relationship was determined by the therapists’ capacity to adapt their relational stance to the needs of the patients (Strupp, 1980a) and, in other cases, by the patients’ respective character structure and way of relating (Strupp, 1980b). The patient’s capacity to form a therapeutic relationship and be involved in productive work following the therapist’s approach interplayed with the therapist’s ability to deal with his or her own personal reactions to the patient’s pathology (Strupp, 1980c). In less successful cases in our study, we found both patients who wanted another therapeutic approach (and lack of negotiation on this issue) and therapists who had difficulties managing their countertransference reactions. To conclude, some patients are not the right patients for the kind of therapy offered by the particular therapist. At least some, if not most therapists are unable to adapt their therapeutic technique and the relational stance to the needs of some patients (cf., Strupp, 1980a). These within-therapist differences indicate that taking a “third position” is most necessary and seems to be most difficult, when early signs of lack of therapeutic progress appear.

In our study, the same therapist could differ with different patients in her capacity to establish a collaborative relationship, to actively use therapeutic interventions, and to promote resolution of therapeutic impasses (Safran et al., 2014). Katz and Hilsenroth (2018) found that encouraging the patients’ emotional experiences, in combination with interpretations of the patients’ interpersonal patterns, was particularly beneficial early in psychodynamic treatment for depression. In line with our findings, Gazzillo et al. (2014) showed that in the good outcome case the therapist used active and correct interventions and at the same time adopted a relational stance. We fully agree with the authors’ conclusion that successful therapeutic work presupposes an interaction between relational and technical focus, especially early in the treatment.

### Strengths and Limitations

One asset of the present study is the focus on contrasting cases within the therapists’ caseload, thereby contributing to our growing knowledge about within-therapist differences. Furthermore, the prospective research design made it possible to explore the participants’ experiences at the outset of treatments that were later classified as successful or less successful, thus it was not necessary to rely solely on retrospective recall. Another advantage is the use of an “objective” quantitative outcome criterion, namely reliable and clinically significant symptom reduction at termination. However, this criterion does not take into consideration other dimensions of improvement, other outcome measures, and improvements as assessed by the therapists or as experienced by the patients. The inductive thematic analysis of interviews opened access the patients’ and their therapists’ unvoiced experiences of therapeutic processes in contrasting cases. On the other hand, the pre-post design and lack of session recordings prevented a closer study of in-session interactions and the development of the therapeutic relationship.

Furthermore, only three therapists were included. As the present study was based on archival data, inviting the therapists to offer their understanding of contrasting cases was not possible. The therapy duration varied between 9 and 46 months. However, we could not see any apparent patterns in this regard. One of the non-improved patients was in the shortest treatment and the deteriorated patient in the longest; in both cases, the termination was initiated by the patient. Moreover, there was no relation between therapy duration and the outcome in the total YAPP sample (Philips et al., 2006).

Our study indicates that a therapist works differently with different patients and that the differences cannot be explained by patient factors alone. Rather than studying the patient and the therapist variables independently, focusing on the patient-therapist dyad as a unit (Silberschatz, 2017) can give us new knowledge that is highly relevant to clinicians. We still need more research, with larger number of therapists treating several patients, and sophisticated methodology to study what in the patient-therapist match and interaction results in contrasting outcomes. We also need studies differentiating between lack of improvement and adverse or harmful effects. Another area for further studies could be contrasting cases of effective and ineffective therapeutic dyads in more directive therapeutic modalities.

### Clinical Implications

Despite its limitations, the present study might have important implications for clinical practice and psychotherapy training. Looking for within-therapist effects, we found both effective and ineffective therapeutic dyads. Our findings suggest that the therapist’s expertise has to be viewed as “case-dependent” (cf., Palmstierna and Werbart, 2013; Werbart et al., 2015, 2018, 2019). In order to prevent suboptimal outcomes, the therapists have to be observant of cases when they, from the beginning, have difficulties in conceptualizing the patient’s problems and their ideas of the coming therapeutic work. Scrutinizing their own and their patients’ way of being together might be more difficult but especially important early in therapy. Negotiating both participants’ ideas of therapeutic goals and tasks might in itself be a mechanism of change. Ongoing metacommunication with the patient about what is going on in the therapeutic relationship might enable therapeutic impasses to be worked through and could prevent unsuccessful treatments (cf., Safran et al., 2014). Such communication can be facilitated by use of formalized feedback instruments (cf., Lambert, 2013; Miller et al., 2015).

In order to find the right interventions, the therapist has to continuously assess the patient’s functioning and be open to reconsidering the initial assessment of the patient’s problematic areas and capabilities (cf., Markowitz and Milrod, 2015). Our study indicates that an important ingredient in psychotherapy training might be guidance on how to balance support and challenge in the therapeutic process and how to adjust to the patient’s needs and relational patterns. This includes training in being attentive to and making active use of the therapist’s positive as well as negative countertransference (Hayes et al., 2011). Furthermore, continuing education has to implement the implications of current research on the therapists’ contributions to negative processes (Castonguay et al., 2010; Hilsenroth et al., 2012). Even the most skilled therapists can learn much from their least successful cases and their own treatment failures.

It is incumbent upon the therapist to differentiate between the therapist’s and the patient’s wishes, fears, and reactions. Doing so involves the therapist intentionally bringing to mind personal experiences that somehow relate to the patient’s suffering, before responding with an exploration of what the patient cannot contain. The therapist’s response has to be “marked” by the difference between the patient’s and the therapist’s perspective, thus making possible a “third position” (Benjamin, 2009). Such a position includes alternating between participation in the patient’s inner world and observation, starting with the self and going to the patient.

## Ethics Statement

The project has been approved by the Regional Research Ethics Committee at the Karolinska Institutet. All participants gave written informed consent in accordance with the Declaration of Helsinki.

## Author Contributions

AW was project leader and principal investigator in the YAPP and in the present study. He planned and designed the work, was responsible for acquisition of all the data included, continuously scrutinized the data analysis, interpretation of results, and early drafting, and prepared the version to be submitted. AA and JH contributed primarily with analysis and interpretation of the data for the work, early drafting, and with critical revision in the later stages of the work. They have also given final approval of the version to be published and agreed to be accountable for all aspects of the work.

### Conflict of Interest Statement

The authors declare that the research was conducted in the absence of any commercial or financial relationships that could be construed as a potential conflict of interest.
